# Should CAH in Females Be Classified as DSD?

**DOI:** 10.3389/fped.2016.00048

**Published:** 2016-05-13

**Authors:** Ricardo González, Barbara M. Ludwikowski

**Affiliations:** ^1^Pediatric Surgery and Urology, Auf der Bult Kinder- und Jugendkrankenhaus, Hannover, Germany

**Keywords:** DSD, congenital adrenal hyperplasia, adrenogenital syndrome, gender assignment

## Abstract

Great controversies and misunderstandings have developed around the relatively recently coined term disorders of sex development (DSD). In this article, we question the wisdom of including XX individuals with congenital adrenal hyperplasia (CAH) in the DSD category and develop arguments against it based on the published literature on the subject. It is clear that females with CAH assigned the female gender before 24 months of age and properly managed retain the female gender identity regardless of the Prader grade. Females with CAH and low Prader grades have the potential for a normal sexual and reproductive life. Those with greater degrees of prenatal androgen exposure (Prader grades IV and V) raised as females also identify themselves as females but experience more male-like behavior in childhood, have a greater rate of homosexuality, and have greater difficulty with vaginal penetration and maintaining pregnancies. Improvement in surgical techniques, better endocrinological, psychological, and surgical follow-up may lessen these problems in the future. Given the fact that the term DSD includes many conditions with problematic gender identity and conflicts with the gender assigned at birth, it may be appropriate to exclude females with CAH from the DSD classification.

A consensus paper in 2006, which reflected the opinion of a group of international experts in the subject, led to the substitution of the term *Intersex* for *disorders of sexual development* (*DSD*). The term *intersex* was considered to be vague and pejorative, and DSD was deemed more appropriate (1). It may be pertinent to go back and analyze the terms.

Intersex refers to a condition in which the appearance of the external genitalia falls somewhere between the two natural sexes, male and female, required for reproduction. Ambiguity of the external genital organs presents many problems for the affected individual, her or his parents, and the medical team involved. The problems begin at birth when the baby is usually declared to be a boy or a girl and a gender-appropriate name is given and continue for the entire life of the affected individual with physical and psychological repercussions.

The term “disorders of sex development” implies that there has been an alteration or deviation (or more politically correctly, a difference) in the normal path of development of the sex organs, which are better referred to as genitals organs involved in sexual activity and reproduction.

The consensus paper included under the umbrella of DSD, conditions such as vaginal agenesis and Klinefelter syndrome (even though the external genitals are normal and pose no doubts regarding gender assignment or identity) but does not include undescended testes or hydrocele, conditions that clearly involve reproductive organs. Also included as DSD was severe hypospadias (without giving a clear definition of what is meant by severe or the underlying cause of the malformation). These and other arbitrary choices give the impression that the conditions grouped as DSD were selected with questionable scientific criteria.

Professionals, particularly physicians, embraced this new terminology readily and with little questioning, perhaps to appear modern, well informed, and sensitive to the current controversies in treatment (2, 3). Lay public and advocacy groups also welcomed the changes, but some patients and families did not approve of the inclusion of congenital adrenal hyperplasia (CAH) as DSD (4).

Controversial issues in DSD relate mostly to cases where there is little evidence for what is the best choice for gender assignment and the timing of corrective surgery if indicated. This is particularly important when the long-term outcome of a given condition is unclear or poor as is the case with many instances of gonadal dysgenesis, male aphallia, ovotesticular DSD, XY cloacal exstrophy (5), and extreme micropenis among others. For those cases, a reconsideration of practices prior to 2006, such as the automatic female gender assignment when the phallus was considered inadequate (6), was probably beneficial. This clinical conduct was based on the theory that the sex of rearing, the appearance of the external genitalia, and the hormonal milieu were the critical factors in determining gender identity (6). However, the clinical application of this hypothesis was often unsuccessful (7) probably because it disregarded the effect of prenatal exposure to androgens, which has an unquestionable influence in future gender-related behavior (8). Nevertheless, the influence of the sex of rearing in future gender identity cannot be completely discarded (9).

On the other hand, the inclusion of CAH in XX individuals in the DSD group may not be appropriate. CAH is one of the most common causes of ambiguous genitals. If one considers that 95% of these patients when properly managed from the endocrinological and surgical points of view will have a female gender identity (10) and potential for sexual activity (11) and normal conception rates, if not always fertility (12), it is difficult to comprehend how controversies about gender assignment and management extended to this condition. A possible explanation is that many diseases, with such as genital appearance at birth, were lumped in the same category leading sometimes to confusion.

This confusion among the lay public is well represented in the 2007 Argentine film *XXY* that won the Critic’s Week award in Cannes in 2007. The protagonist is supposed to represent a girl with CAH who at puberty rebels against her medical treatment, feels uncertain about her gender, and exhibits male sexual behavior. Of course, the karyotype of the title corresponds not to CAH but to Klinefelter’s syndrome. The protagonist is supposed to have undergone five surgical procedures and yet can have intercourse and urinate as a male. If her condition was supposed to be a case of Prader V CAH and still had a functioning phallus at puberty, the nature of the operations she had is perplexing. Besides, she was supposedly taking corticosteroids which she decided to stop, a potentially life-threatening decision in a salt loser. In short, the film propagates erroneous information about CAH such as is often used as an argument against female gender assignment and early corrective surgery.

It is well known that girls with salt-wasting CAH tend to exhibit more tomboyish behavior (13), and in adulthood may have less heterosexual preferences and may be less comfortable with their femininity (14). Nevertheless, in the few cases of gender dysphoria in XX CAH patients reported in the literature, it is not clear if lack of compliance with steroid replacement played a role (10, 15).

One unintended clinical experiment was reported by Woelfle et al. (16). In a survey of German endocrinologists, 16 individuals with XX karyotype with “complete virilization” (but certainly without palpable testes) with an initially missed diagnosis of CAH were raised as males. Six of them were reassigned to female in the first 19 months of life, and they all maintained female gender role. In 10 patients, the correct diagnosis was established after 3 years of age. In seven of them, the male gender was maintained with apparently only one expressing doubts about gender identity. In three cases of late gender, reassignment to female was poorly tolerated in one of them.

Of course, with good neonatal care, Woelfle et al.’s series should have only historical interest since all apparently male newborns with bilateral non-palpable testes should be emergently evaluated for possible CAH with karyotype and hormonal studies.

Thus, it appears that assigning the male gender to Prader V CAH individuals with an XX karyotype diagnosed in the first 2 years of life is not appropriate since they will be certainly infertile, have short stature, and seem to have a greater incidence of gender dysphoria than when assigned the female gender and correctly treated with steroid replacement. Also, it is not clear if the increased incidence of cardiac disease induced by early androgen exposure can be prevented with “good hormonal management” (17).

Some authors argued that given the “unsatisfactory” results (18, 19) of some operations for genital ambiguity, there should be a moratorium on all gender (genital?) surgery in infants (20). The authors argue that there are US and international legal precedents for such action. Mistakenly, they appear to confuse truly “unnecessary operations” or “not medically indicated” such as male or female circumcision (for which the ban makes sense) with procedures done to correct an anatomical defect such as hypospadias repair or correction of CAH, which have the similar medical, ethical, and philosophical foundations as correction of a cleft lip or some forms of craniosynostosis, procedures about which little controversy exists.

In addition, those proposing not to operate or make a female gender assignment in Prader V CAH fail to provide evidence of the medical and psychological outcomes of children brought up without a clear gender assignment who grow up with ambiguous genitals. Introducing such management strategy would represent the type of human experimentation that requires serious medical, ethical, and philosophical consideration.

Why should female gender assignment and early surgical correction in females with CAH be questioned and not male gender assignment and early correction in males with primary hypospadias? Consider the similarities of the two diseases: in both, the chromosomal sex is unequivocal, both can be successfully corrected with surgery leading to potentially normal sexual and reproductive functions, and in both gender, dysphoria is rare (10, 15, 21). Furthermore, reconstructive procedures for both conditions have less than perfect results. The complication rate and need for re-operations for fistulas persistent curvature and imperfect cosmetic appearance in cases of proximal hypospadias (22, 23) are comparable to the need for reoperation at puberty for introital stenosis with old operations in CAH (24). We know that repair of hypospadias in older children carries a higher complication rate that when done in infancy (25–28). The consequences of growing up with ambiguous genitals (i.e., a girl with massive clitoromegaly) are unknown, and evidence that postponing genital surgery until the age of consent will yield better results has not been published (29, 30).

It is worth analyzing some of the reports suggesting poor results of early reconstructive surgery in CAH. For example, Creighton et al. (19) reported unsatisfactory cosmetic results in 41% of patients operated in infancy and that almost all required further surgery either to improve cosmesis or to achieve an adequate introital caliber. These unsatisfactory results may relate to techniques used in London in the 1980s and 1990s. More recently, Lesma et al. (11) reported on long-term follow-up of sexually active women with CAH operated in childhood with the Passerini technique (31). Although there was decreased clitoral sensitivity, all had satisfactory intercourse and orgasm with no differences with normal controls.

Gastaud et al. (32) reported that 18 of 22 women in their report experienced pain during vaginal penetration. The proportion of women with low Prader grades who had vaginal intercourse was similar to the controls (more than 90%) as opposed to high Prader grades (37%). It should be noted that this report includes patients who had clitorectomies and “vaginal pull-through” operations, which have long been abandoned.

Bailez et al. (18, 31) reported in 1992 that 78% of women operated in childhood (also with techniques no longer used) required further surgery for introital stenosis. The procedure required was considered minor, and the success rate was high.

From the above, it can be concluded that CAH females with low Prader grades often achieve normal heterosexual sexual activity and pregnancies. In contrast, Prader grades IV and V although persevering female gender identity tend to have less heterosexual relationships, and those who chose to have them experience greater difficulty with vaginal penetration and maintaining pregnancies. Greater prenatal androgen exposure, poor compliance with hormonal replacement, and inadequate surgical techniques may contribute to these differences. Future efforts should be directed at achieving better anatomical results with the operations performed, better counseling regarding the importance of life long hormonal replacement, and close follow-up till sexual maturation is achieved and thereafter.

It is to be expected that the long-term results and type of follow-up evaluation currently used will yield better long-term results (33–35).

The term DSD carries implications of high rates of dissatisfaction with gender assignment and with surgeries performed to match such gender assignment. This is not the case with well-managed cases of CAH. In fact, in a recent article by a member of the consensus group (1), it is stated that “*Most children with 46,XX karyotype and DSD have congenital adrenal hyperplasia due to 21-hydroxylase deficiency and should be regarded as unchallenged females*” (36). For these reasons, we propose that CAH should not be included in the category of DSD.

One of the risks of misunderstandings in this topic is that the information available to the public is not always objective and unbiased, and often interferes with the relationship between the medical professionals and the families of affected children, and it may also influence politicians and lawmakers to introduce restrictive legislation that will ultimately hurt, rather than help, patients.

In fact, it might be better to stop the use of the term DSD altogether and instead use the specific diagnosis for every given condition.

## Author Contributions

This article was conceived, written, and discussed between the two authors. Both have contributed equal effort to its composition.

## Conflict of Interest Statement

The authors declare that the research was conducted in the absence of any commercial or financial relationships that could be construed as a potential conflict of interest.
